# Complete genome sequence of *Isosphaera pallida* type strain (IS1B^T^)

**DOI:** 10.4056/sigs.1533840

**Published:** 2011-02-20

**Authors:** Markus Göker, David Cleland, Elizabeth Saunders, Alla Lapidus, Matt Nolan, Susan Lucas, Nancy Hammon, Shweta Deshpande, Jan-Fang Cheng, Roxane Tapia, Cliff Han, Lynne Goodwin, Sam Pitluck, Konstantinos Liolios, Ioanna Pagani, Natalia Ivanova, Konstantinos Mavromatis, Amrita Pati, Amy Chen, Krishna Palaniappan, Miriam Land, Loren Hauser, Yun-Juan Chang, Cynthia D. Jeffries, John C. Detter, Brian Beck, Tanja Woyke, James Bristow, Jonathan A. Eisen, Victor Markowitz, Philip Hugenholtz, Nikos C. Kyrpides, Hans-Peter Klenk

**Affiliations:** 1 DSMZ - German Collection of Microorganisms and Cell Cultures GmbH, Braunschweig, Germany; 2 ATCC - American Type Culture Collection, Manassas, Virginia, USA; 3 DOE Joint Genome Institute, Walnut Creek, California, USA; 4 Los Alamos National Laboratory, Bioscience Division, Los Alamos, New Mexico, USA; 5 Biological Data Management and Technology Center, Lawrence Berkeley National Laboratory, Berkeley, California, USA; 6 Oak Ridge National Laboratory, Oak Ridge, Tennessee, USA; 7 University of California Davis Genome Center, Davis, California, USA; 8 Australian Centre for Ecogenomics, School of Chemistry and Molecular Biosciences,The University of Queensland, Brisbane, Australia

**Keywords:** thermophilic, aerobic, filamentous, budding, gliding motility, Gram-negative, phototactic comets, gas vesicles, chemoheterotrophic, hot spring, *Planctomycetaceae*, GEBA

## Abstract

*Isosphaera pallida* (*ex* Woronichin 1927) Giovannoni *et al*. 1995 is the type species of the genus *Isosphaera*. The species is of interest because it was the first heterotrophic bacterium known to be phototactic, and it occupies an isolated phylogenetic position within the *Planctomycetaceae*. Here we describe the features of this organism, together with the complete genome sequence and annotation. This is the first complete genome sequence of a member of the genus *Isosphaera* and the third of a member of the family *Planctomycetaceae*. The 5,472,964 bp long chromosome and the 56,340 bp long plasmid with a total of 3,763 protein-coding and 60 RNA genes are part of the *** G****enomic* *** E****ncyclopedia of* *** B****acteria and* *** A****rchaea * project.

## Introduction

Strain IS1B^T^ (= ATCC 43644) is the type strain of *Isosphaera pallida* which in turn is the type and sole species of the genus *Isosphaera* [1,2]. The genus *Isosphaera* is one out of nine genera in the family *Planctomycetaceae* [3]. The genus name is derived from the Greek adjective *isos*, equal and *sphaera*, a ball, globe, yielding *Isosphaera*, sphere of equal size [4]. The species epithet *pallida* is derived from the Latin adjective *pallida*, pale [1]. Strain IS1B^T^ was isolated from a hot spring in Kah-nee-tah, Oregon, USA [1]. Other closely related strains belonging to the species were isolated from several warm springs in North America [1]. The cells resemble *Isocystis pallida* Worochin 1927 [5] which was previously described as a cyanobacterium and later as a yeast. Here we present a summary classification and a set of features for *I. pallida* strain IS1B^T^, together with the description of the complete genomic sequencing and annotation.

## Classification and features

A representative genomic 16S rRNA sequence of strain IS1B^T^ was compared using NCBI BLAST under default values (e.g., considering only the best 250 hits) with the most recent release of the Greengenes database [6] and the relative frequencies, weighted by BLAST scores, of taxa and keywords (reduced to their stem [7]) were determined. The five most frequent genera were *Isosphaera* (35.4%), *Nostocoida* (26.4%; a genus with *Candidatus* status [8]), *Singulisphaera* (20.4%), '*Isophaera*' (15.9%; a misspelling of *Isosphaera*) and *Planctomyces* (1.9%). The species yielding the highest score was *Candidatus* *Nostocoida limicola* [8]. The five most frequent keywords within the labels of environmental samples which yielded hits were 'skin' (3.9%), 'soil' (3.0%), 'fossa' (2.2%), 'adult/zebrafish' (2.2%) and 'microbi' (1.9%). The two most frequent keywords within the labels of environmental samples which yielded hits of a higher score than the highest scoring species were 'adult, zebrafish' (10.0%) and 'conventionally-rais, digest, gender, germ-fre, gut, habitat, host, mice, micro-biota, mix, pool, recipi, reciproc, select, tract, transplant' (5.0%), i.e. many ties occurred, rendering it difficult to ecologically interpret this outcome.

**Figure 1:**
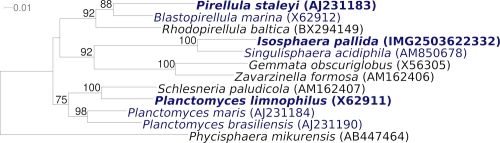
Phylogenetic tree highlighting the position of *I. pallida* relative to the other type strains within the class family *Planctomycetacia*. The tree was inferred from 1,362 aligned characters [9,10] of the 16S rRNA gene sequence under the maximum likelihood criterion [11] and rooted in with members of the class *Phycisphaerae*. The branches are scaled in terms of the expected number of substitutions per site. Numbers above branches are support values from 450 bootstrap replicates [12] if larger than 60%. Lineages with type strain genome sequencing projects registered in GOLD [13] are shown in blue, published genomes in bold [14,15].

Figure 1 shows the phylogenetic neighborhood of *I. pallida* IS1B^T^ in a 16S rRNA based tree. The sequences of the three copies in the genome do not differ from each other, and differ by two nucleotides from the previously published 16S rRNA sequence (AJ231195).

**Figure 2:**
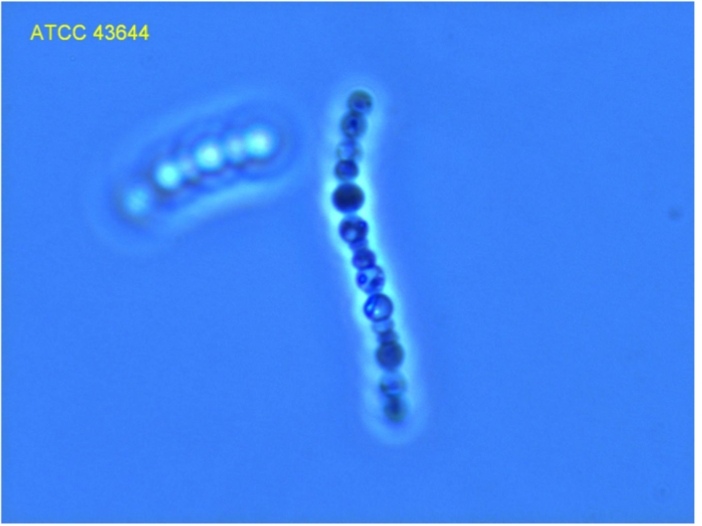
Photomicrograph (1000 x) of *I. pallida* IS1B^T^  (provided by ATCC)

Cells of strain IS1B^T^ are spherical with 2.5 to 3 µm in diameter (Figure 2 and Table 1), with cell growth and division occurring by intercalary budding, resulting in filaments [1]. The cells are salmon-colored (caused by carotenoids), contain gas vesicles and resemble *Isocystis pallida* Worochin 1927 [5]. Ultra-thin sections observed by TEM revealed pit-like ultrastructural features in the cell wall [1,24]. The cells contain numerous pili (not visible in Figure 2) but no flagella, and form motile phototactic “comets” in liquid cultures or on media containing Gelrite^®^ as the solidifying agent [1].

**Table 1:** Classification and general features of *I. pallida* IS1B^T^ according to the MIGS recommendations [16]

**MIGS ID**	**Property**	**Term**	**Evidence code**
	Current classification	Domain *Bacteria*	TAS [17]
		Phylum *Planctomycetes*	TAS [18]
		Class *Planctomycetacia*	TAS [19]
		Order *Planctomycetales*	TAS [3,20-22]
		Family *Planctomycetaceae*	TAS [3,20]
		Genus *Isosphaera*	TAS [1,2]
		Species *Isosphaera pallida*	TAS [1,2]
		Type strain IS1B	TAS [1]
	Gram stain	variable to negative	TAS [1]
	Cell shape	coccoid, chain-forming	TAS [1]
	Motility	motile by gliding	TAS [1]
	Sporulation	not reported	
	Temperature range	40–55°C, thermophile	TAS [1]
	Optimum temperature	41°C	TAS [1]
	Salinity	about 0.1% NaCl	TAS [1]
MIGS-22	Oxygen requirement	obligately aerobic	TAS [1]
	Carbon source	glucose, lactate	TAS [1]
	Energy source	chemoheterotrophic	TAS [1]
MIGS-6	Habitat	algal mat, fresh water	TAS [1]
MIGS-15	Biotic relationship	not reported	
MIGS-14	Pathogenicity	none	NAS
	Biosafety level	1	NAS
	Isolation	hot spring	TAS [1]
MIGS-4	Geographic location	Kah-nee-tah Hot Spring, Oregon,USA	TAS [1]
MIGS-5	Sample collection time	1987 or before	TAS [1]
MIGS-4.1 MIGS-4.2	Latitude Longitude	44.86 -121.20	TAS [1]
MIGS-4.3	Depth	0 m, probably surface waters	NAS
MIGS-4.4	Altitude	not reported	

Evidence codes - IDA: Inferred from Direct Assay (first time in publication); TAS: Traceable Author Statement (i.e., a direct report exists in the literature); NAS: Non-traceable Author Statement (i.e., not directly observed for the living, isolated sample, but based on a generally accepted property for the species, or anecdotal evidence). These evidence codes are from of the Gene Ontology project [23]. If the evidence code is IDA, the property was observed by one of the authors or an expert mentioned in the acknowledgements.

### Chemotaxonomy

Muramic acid and diaminopimelic acid are absent from the cell wall [1,24], like in other members of the *Planctomycetes*. Cells stain Gram-negative but lack an outer membrane [1]. Cells possess a proteinaceous cell wall structure without cysteine, methionine, proline and tryptophan [24]. Ester-linked lipids with predominantly unbranched C_14_ and C_18_ fatty acids, traces of C_18:1_ acids, no hydroxyl-fatty acids [24].

## Genome sequencing and annotation

### Genome project history

This organism was selected for sequencing on the basis of its phylogenetic position [25], and is part of the *** G****enomic* *** E****ncyclopedia of* *** B****acteria and* *** A****rchaea * project [26]. The genome project is deposited in the Genomes OnLine Database [13] and the complete genome sequence is deposited in GenBank. Sequencing, finishing and annotation were performed by the DOE Joint Genome Institute (JGI). A summary of the project information is shown in Table 2.

**Table 2:** Genome sequencing project information

**MIGS ID**	**Property**	**Term**
MIGS-31	Finishing quality	Finished
MIGS-28	Libraries used	Three genomic libraries: one 454 pyrosequence standard library, one 454 PE library (11 kb insert size), one Illumina library
MIGS-29	Sequencing platforms	Illumina GAii, 454 GS FLX Titanium
MIGS-31.2	Sequencing coverage	109.5 × Illumina; 31.2 × pyrosequence
MIGS-30	Assemblers	Newbler version 2.0.00.20-PostRelease-11-05-2008-gcc-3.4.6, Velvet, phrap
MIGS-32	Gene calling method	Prodigal 1.4, GenePRIMP
	INSDC ID	CP002353 (chromosome) CP002354 (plasmid)
	Genbank Date of Release	January 26, 2011
	GOLD ID	Gc01591
	NCBI project ID	32825
	Database: IMG-GEBA	2503538023
MIGS-13	Source material identifier	ATCC 43644
	Project relevance	Tree of Life, GEBA

### Growth conditions and DNA isolation

*I. pallida* IS1B^T^, ATCC 43644, has been in the American Type Culture Collection since July 1987. The culture used  at ATCC to prepare genomic DNA (gDNA) for sequencing was only two transfers away from the original deposit. The purity of the culture was determined by growth in ATCC medium 1962 Broth [27] at 45^o^C under aerobic conditions. Cells were harvested by centrifugation after 72 hours of incubation. The cell pellet exhibited a salmon color. Genomic DNA was extracted from lysozyme-treated cells using a standard CTAB and phenol-chloroform protocol. The purity, quality and size of the bulk gDNA preparation were assessed according to DOE-JGI guidelines. Amplification and partial sequencing of the 16S rRNA gene confirmed the isolate as *I. pallida*. The quantity of the DNA was determined on a 1% agarose using gel mass markers of known concentration supplied by JGI. The average fragment size of the purified gDNA determined to be ~43 kb by pulsed-field gel electrophoresis.

### Genome sequencing and assembly

The genome was sequenced using a combination of Illumina and 454 sequencing platforms. All general aspects of library construction and sequencing can be found at the JGI website [28]. Pyrosequencing reads were assembled using the Newbler assembler version 2.0.00.20-PostRelease-11-05-2008-gcc-3.4.6 (Roche). The initial Newbler assembly, consisting of 36 contigs in 1 scaffold, was converted into a phrap assembly by making fake reads from the consensus [29], to collect the read pairs in the 454 paired end library. Illumina GAii sequencing data (461 Mb) was assembled with Velvet [30] and the consensus sequences were shredded into 1.5 kb overlapped fake reads and assembled together with the 454 data. The 454 draft assembly was based on 172.7 Mb of 454 draft data and all of the 454 paired end data. Newbler parameters are -consed -a 50 -l 350 -g -m -ml 20. The Phred/Phrap/Consed software package [29] was used for sequence assembly and quality assessment in the subsequent finishing process. After the shotgun stage, reads were assembled with parallel phrap (High Performance Software, LLC). Possible mis-assemblies were corrected with gapResolution [28], Dupfinisher, or sequencing cloned bridging PCR fragments with subcloning or transposon bombing (Epicentre Biotechnologies, Madison, WI) [31]. Gaps between contigs were closed by editing in Consed, by PCR and by Bubble PCR primer walks (J.-F.Chang, unpublished). A total of 411 additional reactions and 14 shatter libraries were necessary to close gaps and to raise the quality of the finished sequence. Illumina reads were also used to correct potential base errors and increase consensus quality using a software Polisher developed at JGI [32]. The error rate of the completed genome sequence is less than 1 in 100,000. Together, the combination of the Illumina and 454 sequencing platforms provided 140.7 × coverage of the genome. The final assembly contained 764,175 pyrosequence and 16,816,247 Illumina reads.

### Genome annotation

Genes were identified using Prodigal [33] as part of the Oak Ridge National Laboratory genome annotation pipeline, followed by a round of manual curation using the JGI GenePRIMP pipeline [34]. The predicted CDSs were translated and used to search the National Center for Biotechnology Information (NCBI) nonredundant database, UniProt, TIGRFam, Pfam, PRIAM, KEGG, COG, and InterPro databases. Additional gene prediction analysis and functional annotation was performed within the Integrated Microbial Genomes - Expert Review (IMG-ER) platform [35].

## Genome properties

The genome consists of a 5,472,964 bp long chromosome with a 62% GC content and a 56,340 bp plasmid with 67% GC content (Table 3 and Figures 3a and 3b). Of the 3,823 genes predicted, 3,763 were protein-coding genes, and 60 RNAs; 41 pseudogenes were identified. The majority of the protein-coding genes (59.7%) were assigned with a putative function while the remaining ones were annotated as hypothetical proteins. The distribution of genes into COGs functional categories is presented in Table 4.

**Table 3:** Genome Statistics

**Attribute**	Value	% of Total
Genome size (bp)	5,529,304	100.00%
DNA coding region (bp)	4,671,376	84.48%
DNA G+C content (bp)	3,455,288	62.49%
Number of replicons	2	
Extrachromosomal elements	1	
Total genes	3,823	100.00%
RNA genes	60	1.57%
rRNA operons	3	
Protein-coding genes	3,763	98.43%
Pseudo genes	41	1.07%
Genes with function prediction	2,284	59.74%
Genes in paralog clusters	227	5.94%
Genes assigned to COGs	2,408	62.99%
Genes assigned Pfam domains	2,563	67.04%
Genes with signal peptides	792	20.72%
Genes with transmembrane helices	967	25.29%
CRISPR repeats	3	

**Figure 3a:**
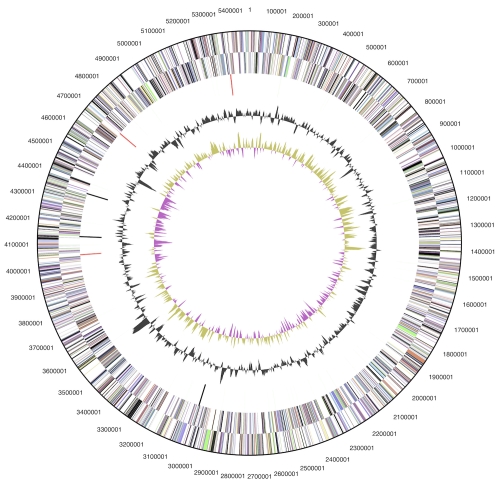
Graphical circular map of the chromosome. From outside to the center: Genes on forward strand (color by COG categories), Genes on reverse strand (color by COG categories), RNA genes (tRNAs green, rRNAs red, other RNAs black), GC content, GC skew.

**Figure 3b:**
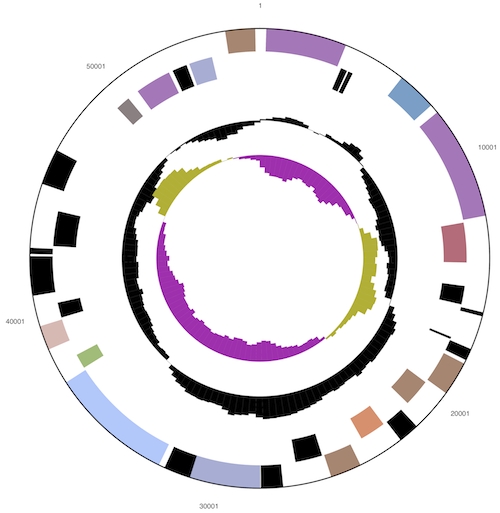
Graphical circular map of the plasmid (not drown to scale with chromosome). From outside to the center: Genes on forward strand (color by COG categories), Genes on reverse strand (color by COG categories), RNA genes (tRNAs green, rRNAs red, other RNAs black), GC content, GC skew.

**Table 4:** Number of genes associated with the general COG functional categories

Code	value	%age	Description
J	138	4.7	Translation, ribosomal structure and biogenesis
A	1	0.0	RNA processing and modification
K	166	5.7	Transcription
L	165	5.7	Replication, recombination and repair
B	1	0.0	Chromatin structure and dynamics
D	24	0.8	Cell cycle control, cell division, chromosome partitioning
Y	0	0.0	Nuclear structure
V	54	1.9	Defense mechanisms
T	187	6.5	Signal transduction mechanisms
M	196	6.8	Cell wall/membrane/envelope biogenesis
N	77	2.7	Cell motility
Z	0	0.0	Cytoskeleton
W	0	0.0	Extracellular structures
U	128	4.4	Intracellular trafficking and secretion, and vesicular transport
O	132	4.5	Posttranslational modification, protein turnover, chaperones
C	157	5.4	Energy production and conversion
G	176	6.1	Carbohydrate transport and metabolism
E	197	6.8	Amino acid transport and metabolism
F	57	2.0	Nucleotide transport and metabolism
H	152	5.3	Coenzyme transport and metabolism
I	83	2.9	Lipid transport and metabolism
P	110	3.8	Inorganic ion transport and metabolism
Q	70	2.4	Secondary metabolites biosynthesis, transport and catabolism
R	435	15.0	General function prediction only
S	190	6.6	Function unknown
-	1,415	37.0	Not in COGs
